# Leveraging extracellular vesicle biology for novel tests and therapeutics for kidney fibrosis

**DOI:** 10.1002/ctm2.70587

**Published:** 2026-04-21

**Authors:** Chin‐Ya Sophie Chiang, Andrew J. Kassianos, Helen Grania Healy, Monica Suet Ying Ng

**Affiliations:** ^1^ Kidney Health Service Royal Brisbane and Women's Hospital Herston Queensland Australia; ^2^ Conjoint Internal Medicine Laboratory, Chemical Pathology Pathology Queensland Herston Queensland Australia; ^3^ Faculty of Health, Medicine and Behavioural Sciences University of Queensland Herston Queensland Australia; ^4^ Institute of Molecular Bioscience University of Queensland Herston Queensland Australia; ^5^ QIMR Berghofer Herston Queensland Australia

**Keywords:** biomarkers, extracellular vesicles, kidney fibrosis, therapeutics

## Abstract

Kidney fibrosis is mechanistically characterised by tissue injury followed by development of a fibrogenic niche. The fibrogenic niche involves chronic inflammation, vascular rarefaction, glomerulosclerosis, tubular atrophy, interstitial accumulation of extracellular matrix which replaces functional kidney parenchyma—culminating in reduced kidney function. Extracellular vesicles are bilipid membrane‐encased nanoparticles that play critical roles as cell‐to‐cell messengers and waste management mechanisms. Extracellular vesciels communication has been demonstrated within the glomerulus, within the tubular compartment and, between tubular and glomerular compartments. Diverse extracellular vesicle cargoes interact with key pathways regulating cell facte and inflammation to mediate kidney fibrosis. Extracellular vesicles cargos can be leveraged as biomarkers to develop minimally invasive tests for kidney fibrosis and pinpoint active pathobiological processes to guide personalised mechanism‐driven care. Mesechymal cell‐derived extracellular vesicles, extracellular vesicles from bioengineered cells, post‐modified extracellular vesicles and suppression of extracellular vesicle uptake have demonstrated promise in preclinical studies for preventing kidney fibrosis. Roadblocks to clinical implementation of extracellular vesicles as biomarkers include lack of standardised collection, measurement, normalisation and data processing protocols; as well as dearth of highly specific biomaker panels. Roadblocks to extracellular vesicle therapeutics include extracellular vesicle heterogeneity, sensitivity to small changes in production parameters, absence of standardised characterisation protocols, technological limitations and knowledge gaps on extracellular vesicle mechanisms of action. This review will contextualise kidney extracellular vesicle pathobiology with principles of clinical implementation to highlight extracellular vesicle potential as minimally invasive biomarkers and novel therapeutics for kidney fibrosis. Strategies to address roadblocks to clinical implementation of extracellular vesicles for kidney fibrosis and chronic kidney disease management will also be explored in this review.

**Key points:**

- Extracellular vesicles (EVs) are bilipid membrane‐encased nanoparticles that play critical roles as cell‐to‐cell messengers and waste management mechanisms.
- EV cargos can be leveraged as biomarkers to develop minimally invasive tests for kidney fibrosis and guide personalised mechanism‐driven care.
- Mesenchymal cell‐derived EVs, EVs from bioengineered cells, post‐modified EVs and suppression of EV uptake have demonstrated promise for preventing kidney fibrosis.

## Graphical abstract

- Extracellular vesicles (EVs) are bilipid membrane‐encased nanoparticles that play critical roles as cell‐to‐cell messengers and waste management mechanisms.
- EVs cargos can be leveraged as biomarkers to develop minimally invasive tests for kidney fibrosis and guide personalised mechanism‐driven care.
- Mesenchymal cell‐derived EVs, EVs from bioengineered cells, post‐modified EVs and suppression of EV uptake have demonstrated promise for preventing kidney fibrosis.

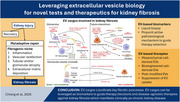

## 1 BACKGROUND

Chronic kidney disease (CKD) is defined as abnormalities of kidney structure or function present for at least 3 months with implications for health.^1^ CKD is mechanistically characterised by oxidative stress and metabolic changes, inflammation, microvascular damage, progressive irreversible nephron loss and decreased regenerative capacity, which culminate in kidney fibrosis.^1^ Kidney fibrosis is manifest by glomerulosclerosis, tubular atrophy, interstitial accumulation of extracellular matrix (ECM) and vascular rarefaction which replaces functional kidney parenchyma—leading to reduced kidney function.^1^ Understanding the pathogenesis of kidney fibrosis is critical to develop biomarkers to stratify CKD progression risk and devise targeted therapeutics to abrogate the inexorable progression to kidney failure.

**FIGURE 1:**
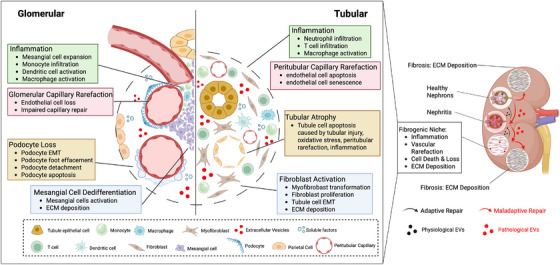
Components of the kidney fibrogenic niche. Kidney fibrosis initiates from fibrogenic niche, foci of fibrogenic cellular events and signalling mediated by soluble factors and extracellular vesicles. The four main events in the fibrogenic niche include inflammation, vascular rarefaction, cell death and ECM deposition. ECM deposition occurs from fibroblast activation in the tubular compartment and mesangial cell activation in the glomerular compartment. Created in BioRender. Chiang, C. (2026) https://BioRender.com/ys26cz7.

Areas of kidney damage can progress to nephron recovery or development of a fibrogenic niche which promotes maladaptive repair and kidney fibrosis.^2^ The fibrogenic niche encompasses four key processes—tissue inflammation, vascular rarefaction, tubular or glomerular atrophy and ECM deposition; and consists of kidney cells, fibroblasts, resident and infiltrating immune cells (Figure 1).^3^, ^4^ In the glomerulus; mesangial cells, podocytes and parietal epithelial cells are the primary kidney contributors to glomerulosclerosis via podocytopenia, podocyte foot process effacement, mesangial expansion, podocyte epithelial–mesenchymal transition (EMT), and parietal cell activation.^5^ Mesangial cells and parietal epithelial cells are the two main sources of ECM. In kidney tubules, proximal tubule epithelial cells (PTECs) are the main contributor of tubulointerstitial fibrosis via PTEC death, PTEC senescence, tubular EMT and fibroblast activation.^6^ Fibroblasts and myofibroblasts are the two main sources of ECM. Paracrine and autocrine signalling between cellular components occurs via ECM networks, cytokines, metabolites, hormones and extracellular vesicles (EVs), which propagate tissue injury and orchestrate fibrosis.^7^

**FIGURE 2:**
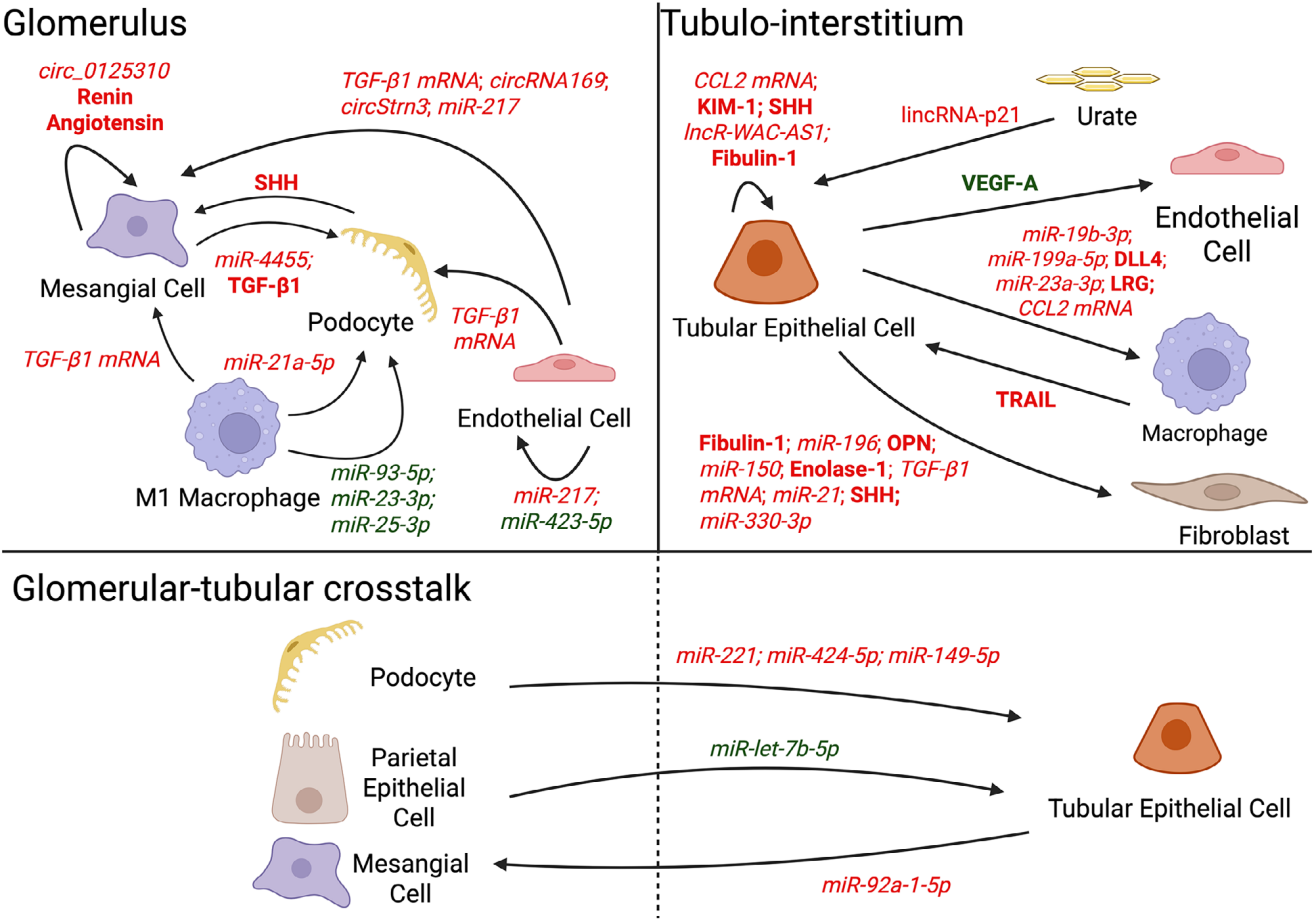
Fibrogenic communication between cells in kidney. Biomolecules carried by extracellular vesicles (EVs) secreted by kidney cells mediate the progression of kidney fibrosis within the glomerulus and tubulointerstitium respectively. EVs also carry out glomerulotubular crosstalk by exchanging biomolecules to mediate the development of kidney fibrosis. Renoprotective cargoes are shown in green and pro‐fibrotic cargoes are shown in red. Proteins are highlighted in bold. DNAs and RNAs are in italicised font. CCL2, CC‐chemokine ligand 2; Circ, circular; DLL4, delta‐like canonical notch ligand 4; KIM‐1, kidney injury molecule‐1; LRG‐1, leucine‐rich α‐2 glycoprotein 1; miR, micro RNA; mRNA, messenger RNA; OPN, osteopontin; SHH, sonic hedgehog; TGF‐β1, transforming growth factor beta 1; TRAIL, tumour necrosis factor‐related apoptosis‐inducing ligand; VEGF‐A, vascular endothelial growth factor‐A. Created in BioRender. Chiang, C. (2026) https://BioRender.com/rmsevrs.

EVs are lipid bilayer‐encased nanoparticles released by all living cells that play critical roles in cell‐to‐cell communication, waste removal, cell maturation, adaptation to environmental changes and activation of blood clotting.^8^ EV cargos include lipids, carbohydrates, metabolites, proteins, ribonucleic acids (RNAs) and deoxyribonucleic acids (DNAs).^9^ EVs can be transmitted across the glomerulus, tubulointerstitium, peritubular capillaries and urinary space to facilitate crosstalk between mesangial cells, podocytes, immune cells, tubular epithelial cells and fibroblasts (Figure 2). EV cargos mediate fibrosis by regulating cell fate (e.g., macrophage polarisation, apoptosis, EMT) via key pathways such as transforming growth factor β1/suppressor of mothers against decapentaplegic (TGF‐β1/SMAD),^10^, ^11^ wingless‐related integration site/β‐catenin (Wnt/β‐catenin),^12^ phosphoinositide 3‐kinase/protein kinase B (PI3K/Akt),^13^ protein kinase B/mechanistic target of rapamycin (Akt/mTOR),^14^ NOTCH^15^ and Janus kinase/signal transducer and activator of transcription kinases (JAK/STAT).^16^ This review will discuss how EVs mediate processes within the fibrogenic niche; and demonstrate how EVs can be leveraged for CKD management as: non‐invasive tests to quantify kidney fibrosis, indicators of active pathways to inform personalised therapy selection, and targeted therapeutics.

## 2 EVS AS KEY DRIVERS OF THE FIBROGENIC NICHE

### 2.1 Tissue injury and inflammation

Under injurious conditions, kidney cells release EVs which propagate waves of cell death and inflammation (Table 1). These EVs mediate cell‐to‐cell communication within glomerular and tubular compartments (Figure 2). In diabetic kidney disease, mesangial cells release EVs containing TGF‐β1 which induce podocyte apoptosis and reduce podocyte adhesion via the PI3K/AKT pathway.^13^ In an in vitro model of immunoglobulin A nephropathy, mesangial cells treated with aberrant immunoglobulin A1 upregulate release of EVs containing *miR‐4455* which downregulate Unc‐51 like autophagy activating kinase 2 (ULK2) to inhibit autophagy and induce podocyte apoptosis.^17^ Puromycin‐induced and lipopolysaccharide (LPS)‐induced podocytes release EVs which increase parietal cell proliferation, activation and motility—albeit to different extents, highlighting the presence of disease‐specific EV signalling and the necessity of investigating disease‐specific approaches to prevent maladaptive repair.^18^

**TABLE 1:** Roles of extracellular vesicle (EV) cargoes in fibrogenic niche.

Source cell	Target cell	Cargo	Disease	Target/pathway	Outcome	Refs.
**Tissue injury and inflammation**						
Mesangial cells	Podocyte	miR‐4455	IgA nephropathy	ULK2	Inhibit autophagy + activate apoptosis	^17^
		TGF‐β1	Diabetic kidney disease	PI3K/AKT	Cell death/detachment	^13^
Mesangial cells	Mesangial cells	Renin, angiotensinogen	Diabetic kidney disease	Renin–angiotensin system	Increase pro‐fibrotic cytokine expression, increase fibronectin expression	^20^
Podocytes	Parietal epithelial cells	NR	Puromycin‐induced, LPS‐induced podocytes, Crescentic glomerulonephritis	NR	Altered cell proliferation and activation	^18^
Podocytes	CD4+ T cells	NR	LPS‐induced podocytes	NR	Immune cells differentiation	^21^
Macrophage	Mesangial cells	TGF‐β1 mRNA	Diabetic kidney disease	TGF‐β1	Cell proliferation/ECM deposition	^23^
M1 macrophage	Podocytes	miR‐21a‐5p	Diabetic kidney disease	TNPO1	Cell injury	^24^
M2 macrophage	Podocytes	miR‐93‐5p	Diabetic kidney disease	TLR4	Attenuate podocyte apoptosis	^25^
		miR‐23‐3p	Diabetic kidney disease	KLF3/STAT3	Attenuate podocyte injury	^27^
		miR‐25‐3p	Diabetic kidney disease	DUSP1	Activate autophagy in podocyte	^26^
Tubular epithelial cells	Tubular epithelial cells	lncR‐WAC‐AS1	Ischaemia–reperfusion injury	GFPT1/ hexosamine biosynthesis pathway	Propagate ferroptotic PTEC injury	^29^
		KIM‐1	Ischaemia–reperfusion injury	NR	Increase expression of inflammatory cytokines (MCP‐1, TNF‐α, IL‐6)	^30^
Tubular epithelial cells	Macrophage	miR‐19b‐3p	LPS‐induced AKI, Adriamycin‐induced CKD, Diabetic kidney disease	SOCS‐1/STAT3	Macrophage activation and infiltration	^32^
		miR‐199a‐5p	Albumin‐induced TECs, Diabetic kidney disease	Klotho	Macrophage activation and infiltration	^33^
		DLL4	Diabetic kidney disease	NOTCH	Macrophage activation	^15^
		miR‐23a‐3p	Ischaemia–reperfusion injury, Diabetic kidney disease	EGF1	Macrophage activation	^35^, ^156^
		CCL2 mRNA	Albumin‐induced TECs, IgA nephropathy	DUSP6	Macrophage activation	^34^, ^36^
		LRG1	Diabetic kidney disease	TGF‐β1	Macrophage activation	^37^
Macrophage	Tubular epithelial cells	TRAIL	Diabetic kidney disease	NR	Apoptosis	^37^
**Vascular rarefaction**						
Endothelial cell	Mesangial cells	miR‐217	Diabetic kidney disease	Sirt1/HIF‐1α	Promote pro‐inflammatory and pro‐fibrotic cytokine expression	^42^
Endothelial cell	Endothelial cells	miR‐217	Long‐term cell culture, Doxorubicin‐treated	SIRT1/p53	Senescence	^43^, ^44^
		miR‐423‐5p	Ischaemia–reperfusion injury	HIF1A/VEGF	Enhanced resistance to apoptosis, increased migration and angiogenesis	^46^
Tubular epithelial cells	Endothelial cells	VEGF‐A	Ischaemia–reperfusion injury	VEGF	Capillary proliferation	^45^
**Glomerular and tubular atrophy**						
Endothelial cell	Podocytes	TGF‐β1 mRNA	Diabetic kidney disease	Wnt/β catenin	Epithelial–mesenchymal transition	^12^
PTEC	PTEC	Fibulin‐1	Diabetic kidney disease	NR	Epithelial–mesenchymal transition	^47^
**ECM deposition**						
Endothelial cell	Mesangial cells	TGF‐β1 mRNA	Diabetic kidney disease	TGF‐β1	Cell activation/ECM deposition	^20^, ^50^
		circRNA169	Diabetic kidney disease	NR	Cell activation/ECM deposition	^51^
		circStrn3	Diabetic kidney disease	NR	Cell activation/ECM deposition	^51^
Mesangial cells	Mesangial cells	circ_0125310	Diabetic kidney disease	IGF1R	Cell proliferation	^4^
Podocytes	Mesangial cells	SHH	Angiotensin II‐treated podocytes	NR	Stimulate mesangial cell activation and proliferation, increased ECM protein expression	^52^
PTEC	Fibroblast	Enolase‐1	Diabetic kidney disease	NR	Cell activation	^54^
		miR‐196	Diabetic kidney disease	STAT3/SOCS2	Cell activation/ECM deposition	^55^
		OPN	Chronic kidney disease, ischaemia–reperfusion injury	CD44	Cell activation and proliferation	^56^
		miR‐150	Ischaemia–reperfusion injury, unilateral ureteric obstruction	NR	Cell activation/ECM deposition	^58^, ^157^
		TGF‐β1 mRNA	Hypoxia	TGF‐β1	Cell activation/ECM deposition	^6^
		miR‐21	Unilateral ureteric obstruction	AKT; TGF‐β1/SMAD	Cell activation/ECM deposition	^10^, ^11^
		SHH	Unilateral ureteral obstruction, ischaemia–reperfusion injury, 5/6 nephrectomy	SHH/GLI	Cell activation/ECM deposition	^10^
		miR‐330‐3p	Uric acid	CREBBP	ECM deposition	^61^
PTEC	Tubular epithelial cells	SHH	Unilateral ureteral obstruction, ischaemia–reperfusion injury, 5/6 nephrectomy	WNT/ β‐catenin; NOTCH	Epithelial–mesenchymal transition	^10^
PTEC	Mesangial cells	miR‐92a‐1‐5p	Diabetic kidney disease	RCN3 mRNA	Epithelial–mesenchymal transition	^67^
Podocytes	PTEC	miR‐221	Diabetic kidney disease	WNT/ β‐catenin	Cell dedifferentiation	^64^
		miR‐424‐5p	Puromycin‐induced	NR	Cell dedifferentiation	^63^
		miR‐149‐5p	Puromycin‐induced	FOXM1	Cell dedifferentiation	^63^
Parietal epithelial cells	Tubular epithelial cells	miR‐let‐7b‐5p	Unilateral ureteral obstruction	TGF‐β1 mRNA and ARID3a mRNA	Reduce inflammation and fibrosis	^68^

Abbreviations: ARID3a, AT‐rich interaction domain 3a; CCL2, C–C motif chemokine ligand 2; CD4+ T cells, cluster of differentiation 4 positive T cells; circRNA, circular RNA; DLL, delta‐like ligands; DUSP1, dual specificity phosphatase 1; DUSP6, dual specificity phosphatase 6; ECM, extracellular matrix; EGR1, early growth response 1; FOXM1, forkhead box protein M1; GFPT1, glutamine–fructose‐6‐phosphate transaminase 1; GLI, glioma‐associated oncogene homolog; HIF‐1α, hypoxia‐inducible factor 1α; IL‐6, interleukin‐6; KIM‐1, kidney injury molecule 1; KLF3/STAT3, Krüppel‐like factor 3/signal transducer and activator of transcription 3; lncR‐WAC‐AS1, long non‐coding RNA WAC antisense RNA 1; LRG1, leucine‐rich alpha‐2‐glycoprotein 1; M1 macrophages, classically activated macrophages; M2 mcrophages, alternatively activated macrophages; MCP‐1, monocyte chemoattractant protein‐1; miR, microRNA; mRNA, messenger RNA; NOTCH, neurogenic locus notch homolog protein; NR, not recorded; OPN, osteopontin; PI3K/ AKT, phosphatidylinositol 3‐kinase/protein kinase B; PTEC, proximal tubular epithelial cells; RCN3, reticulocalbin‐3; Refs., references; SHH, sonic hedgehog; SIRT1, sirtuin 1; SMAD, small mothers against decapentaplegic; SNAIL, zinc finger protein SNAI1; SOCS‐1, suppressor of cytokine signalling 1; SOCS2, suppressor of cytokine signalling 2; TGF‐β1, transforming growth factor beta‐1; TLR4, toll‐like receptor 4; TNF‐α, tumour necrosis factor‐α; Tnpo1, transportin 1; VEGF, vascular endothelial growth factor; Wnt, wingless‐related integration site.

Injured glomerular podocytes, mesangial and parietal epithelial cells secrete chemokines and activate NLR family pyrin‐domain containing 3 (NLRP3) in kidney resident cells. This process induces recruitment of immune cells and pro‐inflammatory cytokine release—contributing to further glomerular injury and activation of downstream pro‐fibrotic processes.^19^ Renin–angiotensin system components from glucose‐induced mesangial cell‐derived EVs act on surrounding mesangial cells to increase pro‐fibrotic cytokine and fibronectin expression.^20^ LPS‐induced podocytes release EVs which stimulate the differentiation of naïve cluster of differentiation (CD)4 T cells to T helper 17 cells (Th17) and T regulatory cells (Treg). Elevated Th17:Treg ratios have been associated with albuminuria and tubulointerstitial fibrosis in a mouse model of diabetic nephropathy.^21^, ^22^ Macrophages cultured at high glucose concentrations release *TGF‐β1* messenger RNA (mRNA) positive EVs that induce mesangial cell proliferation and ECM deposition in vitro and, mesangial cell expansion and kidney fibrosis in vivo.^23^ M1 macrophages release *miR‐21a‐5p* positive EVs which induce podocyte injury via increased transportin 1 (Tnpo1) expression.^24^ Conversely, M2 macrophages release reno‐protective EVs containing *miR‐93‐5p*, *miR‐23‐3p* and *miR‐25‐3p* which act on toll‐like receptor 4 (TLR4), Krüppel‐like factor 3 (KLF3)/STAT3 and dual specificity phosphatase 1 (DUSP1) pathways to attenuate podocyte cell death.^24^, ^25^, ^26^, ^27^

EV signalling in tubulointerstitial injury and inflammation have primarily focussed on interactions involving tubular epithelial cells and macrophages. Various disease‐specific EV cargos activate macrophages and enact downstream inflammation. EVs released by hypoxic kidney cells enter tubular cells via kidney injury molecule 1 (KIM‐1) phosphatidylserine interaction and release microRNAs (miRNAs) and long non‐coding RNAs (lncRNAs) which propagate ferroptotic PTEC injury and induce macrophage expression of inflammatory cytokines.^28^, ^29^, ^30^ Albumin‐induced tubular epithelial cells release EVs containing *miR‐19b‐3p* and *miR‐199a‐5p* which promote M1 macrophage polarisation via inhibition of suppressor of cytokine signalling 1 (SOCS‐1) gene transcription and downregulation of Klotho translation.^31^, ^32^, ^33^ High glucose‐induced tubular epithelial cells release Delta‐like 4 (DLL4)‐rich EVs which stimulate M1 macrophage polarisation by enhancing NOTCH signalling.^15^ Hypoxia‐induced or albumin‐induced tubular epithelial cells release EVs with *miR‐23a* and C–C motif chemokine ligand 2 (*CCL2*) mRNA, respectively, which activate macrophages and promote tubulointerstitial inflammation and fibrosis.^34^, ^35^, ^36^ Lipotoxic tubular epithelial cells release EVs enriched with leucine‐rich α2‐glycoprotein 1 (LRG1) which bind TGF‐βR1 to activate macrophages.^37^ These macrophages subsequently release EVs containing tumour necrosis factor (TNF)‐related apoptosis‐inducing ligand (TRAIL) which increase tubular epithelial cell apoptosis—further amplifying tubular injury.^37^ To date, cell‐to‐cell EV communication mechanisms have primarily been verified using co‐culture experiments which restricts the cell combinations that can be studied and fidelity to the complex multi‐cellular microenvironments. Advances in EV analytics (e.g., in situ EV imaging, EV flow cytometry) offers the potential for high‐throughput assessment of single EVs from multiple cells at once—however, universal markers for EV identification and markers for EV source and targets are urgently needed to distinguish the breadth of cell‐to‐cell EV communication in kidney fibrosis (Table 4).^2^, ^38^, ^39^

### 2.2 Vascular rarefaction

Reduction in vascular density, also known as vascular rarefaction, occurs in the glomerulus and peritubular capillaries in response to tissue injury. Vascular rarefaction induces chronic hypoxia and repeated ischaemic injury which leads to maladaptive repair of injured tubular cells and subsequently tubulointerstitial fibrosis.^40^ Increased urinary endothelial EVs (plasmalemma vesicle‐associated protein [PL‐VAP]+, CD31+ or CD144+) in people with loss of peritubular capillaries in the context of hypertension suggests a role for EVs in propagating vascular rarefaction.^41^ Senescent endothelial cells release EVs containing *miR‐217* which can propagate endothelial senescence, whilst promoting pro‐inflammatory and pro‐fibrotic cytokine expression (e.g., connective tissue growth factor [CTGF], endothelin‐1, fibronectin, TGF‐β1 and vascular endothelial growth factor [VEGF]) in mesangial cells.^42^, ^43^, ^44^ Tubular epithelial cell and endothelial EVs can also protect against vascular rarefaction. EVs released from the basolateral surface of tubular epithelial cells are enriched with VEGF‐A and promote peritubular capillary proliferation and inflammatory cytokine release (e.g., TNF‐α, CCL2, vascular cell adhesion molecule 1 [VCAM1], intercellular adhesion molecule 1 [ICAM1]) after ischaemic stress.^45^ EV‐based VEGF‐A is required for endothelial cell uptake of tubular epithelial cell EVs, with VEGF‐A positive EVs but not soluble VEGF‐A mediating endothelial proliferation.^45^ During acute kidney injury, endothelial cells release EVs containing *miR‐423‐5p* which correlates with increased resistance to apoptosis, migratory capacity, *VEGFA* mRNA levels and enhanced angiogenesis; ultimately reducing vascular rarefaction and kidney fibrosis.^46^ Further studies are urgently needed to elucidate the tubulo‐endothelial and immuno‐endothelial EV communication driving vascular rarefaction and amplifying downstream fibrotic processes.

### 2.3 Glomerular and tubular atrophy

Glomerular and tubular atrophy is associated with EMT, senescence and cell death. These events collectively trigger inflammatory responses leading to kidney fibrosis. In a diabetic kidney disease model, endothelial cells undergo endothelial–mesenchymal transition and release EVs which trigger podocyte EMT and barrier dysfunction. This is achieved via EV‐based transfer of *TGF‐β1* mRNA which activates Wnt/β‐catenin signalling in podocytes.^12^ Fibulin‐1 from high glucose‐induced PTEC EVs mediate PTEC EMT.^47^ Circulating EVs from people with antibody‐mediated rejection induce tubular senescence and EMT, potentially via *miR‐604*, *miR‐515‐3p*, *miR‐let‐7d‐5p* or *miR‐590‐3p*, which are all upregulated in EVs from the antibody‐mediated rejection group compared to transplant controls.^48^ The majority of studies linking EVs to glomerular and tubular atrophy have been completed using diabetic kidney disease models. Further studies are required to determine if EMT‐mediating EVs cargos are conserved regardless of initial mechanism of injury—offering the potential for disease‐agnostic CKD therapy.

### 2.4 ECM deposition

Excessive ECM is synthesised by activated mesangial and parietal epithelial cells in the glomerulus and fibroblasts in the tubulointerstitium.^49^ In diabetic kidney disease, glomerular endothelial cells release EVs containing *TGF‐β1* mRNA, *circRNA169* and *circStrn3* which activate mesangial cells and ECM deposition.^20^, ^50^, ^51^ Mesangial cells secrete EVs carrying circular RNA (*circ_0125310)* which sponge *miR‐422a* and, upregulate IGF1R and p38 expression to promote mesangial cell proliferation in a mouse model of diabetic kidney disease.^4^ Angiotensin II‐treated podocytes release EVs containing sonic hedgehog (SHH) which stimulate mesangial cell activation leading to increased ECM protein expression—contributing to glomerulosclerosis.^52^

In the tubulointerstitial compartment, EVs play critical roles in fibroblast activation, leading to increased α‐smooth muscle actin (α‐SMA), vimentin and fibronectin expression.^53^ High glucose‐induced PTECs release EVs enriched with Enolase‐1 which activate fibroblasts in vitro.^54^ In the presence of aldosterone or β‐catenin, PTECs secrete EVs that carry *miR‐196* or osteopontin (OPN) which promote fibroblast activation and ECM deposition.^55^, ^56^ Moreover, intravenous injection of *miR‐196* positive EVs from the kidney cortex of aldosterone‐treated mice into healthy mice promotes kidney fibrosis.^55^ Hypoxic PTECs secrete EVs carrying *miR‐150*, *miR‐150‐5p* and *TGF‐β1* mRNA that activate fibroblasts and promote ECM deposition.^6^, ^57^, ^58^ In response to TGF‐β1 stimulation, PTECs release EVs containing *miR‐21* and SHH protein which also promote ECM deposition.^10^, ^59^ *miR‐21* is a central regulator of kidney fibrosis by targeting: (i) peroxisome proliferator‐activator receptor‐α (PPAR‐α) to increase generation of reactive oxygen species; (ii) phosphatase and TENsin homolog (PTEN) to enhance Akt pathway activation and subsequent EMT; (iii) matrix metalloproteinase 9 (MMP‐9) and reversion‐inducing cysteine‐rich protein (RECK) to enhance ECM deposition; (iv) SMAD7 to increase phosphorylation of SMAD3 and TGF‐β1 signalling; and (v) dimethylarginine dimethylaminohydrolase 1 (DDHA1) to increase asymmetric dimethylarginine (ADMA) accumulation, vascular rarefaction and further tubular injury.^11^ SHH activates fibroblasts via the canonical SHH/glioma‐associated oncogene homolog (GLI) signalling pathway.^60^ SHH can also upregulate Wnt2b, Wnt5a and Snail family transcriptional repressor 1 (SNAIL1) in the Wnt/β‐catenin pathway and Jaggad2 (JAG2) in the NOTCH pathway to facilitate EMT in tubular epithelial cells.^60^ The kidney pro‐fibrotic effect of *miR‐21* and SHH is demonstrated in vivo by intravenous injection of EVs from TGF‐β1‐treated PTECs with *miR‐21* and SHH inhibitor respectively into unilateral ureteric obstruction (UUO) mice.^10^, ^59^ Uric acid stimulated proximal tubule cells release EVs with *miR‐330‐3p* which inhibit translation of cyclic adenosine monophosphate‐responsive element‐binding protein (CREBBP) in fibroblasts to exacerbate kidney fibrosis in an adenine mice model of CKD.^61^ Shortcomings of current approaches to verify the in vivo role of EV cargos in kidney fibrosis include limited capacity to recapitulate physiological EV transport and dose, while intravenously delivered EVs may not accurately simulate endogenous autocrine and paracrine EV transport in the kidney. Knowledge of EV concentrations in the kidney could also be improved by isolation/imaging of EVs in kidney tissue. Furthermore, EVs tend to be tested between specific cell type pairs and there is limited investigation regarding the selective uptake of EV subsets by different cell types. These limitations could be addressed by advanced imaging techniques (e.g., intravital microscopy through an imaging window placed above the kidney in animal models^2^) to allow direct observation of labelled EVs in living animals in real‐time.

## 3 TUBULOGLOMERULAR CROSSTALK IN THE FIBROGENIC NICHE

EVs facilitate bidirectional crosstalk between the glomerular and tubulointerstitial compartments that amplify local injury and coordinate fibrosis across the whole nephron. EVs from injured podocytes contain miRNAs which trigger tubular cell dedifferentiation (i.e., *miR‐221*) and apoptosis (i.e., *miR‐424‐5p*, *miR‐149‐5p*).^62^, ^63^, ^64^, ^65^ *miR‐221*‐containing EVs mediate PTEC injury via Wnt/β‐catenin signalling, with inhibition of *miR‐221* abrogating PTEC injury in streptozotocin‐induced mice.^64^ These events subsequently promote tubular cell EMT and ECM deposition via the TGF‐β receptor‐mediated activation of SMAD3 and CD36‐associated stimulation of p38 MAPK.^66^ Moreover, EVs from high glucose‐stimulated PTECs contain *miR‐92a‐1‐5p* which trigger endoplasmic reticulum stress and induce mesangial cell injury, EMT and ECM deposition.^67^ In particular, *miR‐92a‐1‐5p* directly binds and modulates *RCN3* mRNA translation to induce endoplasmic reticulum stress via calretriculin (CALR) and mesencephalic astrocyte‐derived neurotrophic factor (MANF).^67^ EVs cargos can also protect against kidney inflammation and fibrosis. In obstructive kidney disease, EVs from parietal epithelial cells are enriched for *miR‐let‐7b‐5p* which binds and regulates the translation of *TGF‐β1* and *ARID3a* mRNA in tubular epithelial cells.^68^

## 4 EV CARGO AS BIOMARKERS FOR MINIMALLY INVASIVE TESTS OF KIDNEY FIBROSIS

The standard‐of‐care for kidney fibrosis quantification is kidney biopsy which is invasive, susceptible to sampling error and, carries significant procedural risks of bleeding, pain at biopsy site and infections.^69^ Kidney biopsies require specialised equipment and operators to perform and interpret results—reducing access in resource‐limited settings and rural/regional/remote areas.^70^ These factors limit capacity to serially track kidney fibrosis; the irreversible histological manifestation of CKD. While the presence of kidney fibrosis can be inferred from the irreversibility of serum creatinine rises, this method requires a period of observation—leading to treatment delays. Quantifying kidney fibrosis is critical to identify patients most likely to respond to therapy and inform patient counselling discussions. Minimally invasive methods to quantify kidney fibrosis are urgently needed to improve access to care in resource‐limited settings, facilitate timely treatment selection and enhance patient education.

**FIGURE 3:**
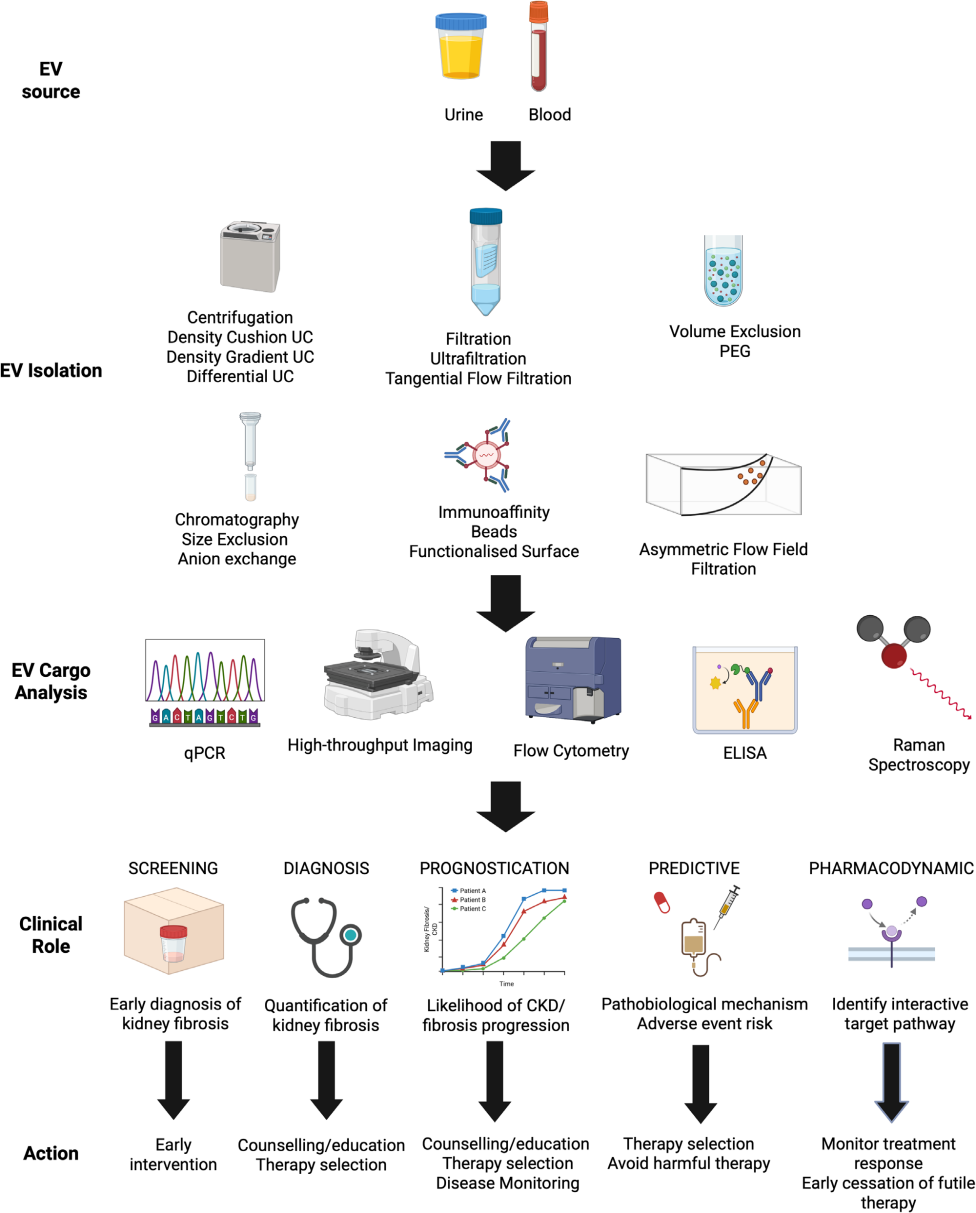
Workflow for integration of extracellular vesicle (EV)‐based tests for kidney fibrosis in chronic kidney disease (CKD) care. Biosample collection such as urine and blood would be collected per current standard processes. There are a range of EV isolation techniques that separate EVs based on size (filtration, differential ultracentrifugation (UC), size exclusion chromatography, asymmetric flow field flow fractionation), density (density gradient ultracentrifugation, density cushion ultracentrifugation), charge (anion exchange) and surface molecules (immuno‐affinity). Other methods include volume exclusion with polyethylene glycol (PEG).These methods are described in further detail by Hendrix et al.^188^ Biased molecular quantification methods can be used for RNA/DNA (qPCR) and protein (high‐throughput immuno‐imaging, flow cytometry, enzyme‐linked immunosorbent assay [ELISA], Raman spectroscopy) analysis. Unbiased methods are not shown here as they are challenging to implement between laboratories and mainly used in hypothesis‐generating research studies. EV cargos can then be used for screening, diagnosis, prognostication, predictive and pharmacodynamic capacities to facilitate early intervention, education and counselling, therapy selection, avoidance of harmful and futile therapies and monitor therapy response. Created in BioRender. Chiang, C. (2026) https://BioRender.com/l7m0w1q.

Urine and blood EVs are an optimal solution for minimally invasive testing due to the durability of their cargos and critical roles in kidney fibrosis pathobiology. These features enable EVs to be integrated into diagnostic, prognostic, predictive and pharmacodynamic components of the clinical CKD workflow (Figure 3). EVs cargos are more robust compared to free molecules owing to their containment in a bilayer lipid membrane and correlate better with disease phenotype.^71^ uEVs stored with ethylenediaminetetraacetic acid (EDTA) preservative are stable at room temperature for up to 8 days enabling self‐sampling at home and mail‐in tests.^72^ Moreover, urine are enriched with kidney EVs from all segments of the nephron, making uEVs invaluable for providing high‐resolution panoramic data on homeostatic and pathogenic processes in the kidney.^73^ Urine and blood EVs cargos have been correlated to kidney fibrosis measured by interstitial fibrosis and tubular atrophy across diverse CKD aetiologies (Table 2).

**TABLE 2:** Extracellular vesicle (EV) cargos as biomarkers for kidney fibrosis.

Correlation to fibrosis	Biomolecule	Kidney diseases (biofluid)	Sample size	Pathway	Role in other diseases	Refs.
Positive	miR‐19	Kidney fibrosis (B)	97	PTEN/Akt	↑ with stroke severity; ↓ in pancreatic cancer, head and neck cancer	^158^, ^159^, ^160^, ^161^
	miR‐21	Chronic kidney disease (U)—diabetic nephropathy, focal segmental glomerulosclerosis, IgA nephropathy, membranous nephropathy, mesangial proliferative renal disease, minimal change disease, amyloid nephropathy	55	TGF‐β1/SMAD	↑ in prostate cancer, bladder cancer, gastric cancer	^16^, ^74^, ^76^, ^77^, ^78^, ^87^
		Lupus nephritis (U)	65			
		Kidney transplant donors (U)	109			
		Kidney transplants (P)	52		↑ in non‐small cell lung cancer, peritoneal recurrence of gastric cancer, breast cancer	^75^, ^162^, ^163^, ^164^
	miR‐29c	Kidney transplant donor (U)	109	Wnt/β‐Catenin	–	^87^
	miR‐146a	Lupus nephritis (U)	47	NF‐κB/TRAF6	↓ in hypertensive nephropathy, ↑ in active lupus nephritis	^165^, ^166^, ^167^
	miR‐150	Lupus nephritis (U)	65	JAK/STAT	↑ in clear cell renal cell carcinoma	^16^, ^168^
	miR‐205	Kidney transplant donor (U)	109	SNHG16/HDAC5	↑ in bladder urothelial carcinoma	^87^, ^169^
	miR‐330‐3p	Chronic kidney disease (U)	60	CREBBP	–	^61^
	N‐osteopontin	Chronic kidney disease (U)	213	CD44	–	^56^
	hsa_circ_0008925	Chronic kidney disease (U)—IgA nephropathy, membranous nephropathy, minimal change disease, focal segmental glomerulosclerosis, diabetic nephropathy, intracapillary proliferative glomerulonephritis, mesangial proliferative glomerulonephritis, membranoproliferative glomerulonephritis, crescentic glomerulonephritis, lupus nephritis	89	SRSF6	–	^170^, ^171^
	PL‐VAP, CD31, CD144	Hypertension (U)	38	Vascular rarefaction	–	^41^
Negative	miR‐19	Kidney transplant donor (U)	109	PTEN/Akt	↑ with ↓ risk biochemical recurrence of prostate cancer	^87^
	miR‐29a	Chronic kidney disease (U)—diabetic nephropathy, focal segmental glomerulosclerosis, IgA nephropathy, membranous nephropathy, mesangial proliferative glomerulonephritis	39	MMP	↑ in IgA nephropathy, metabolic syndrome, early diabetic kidney disease	^16^, ^84^, ^85^, ^172^, ^173^, ^174^
	miR‐29c	Chronic kidney disease (U)—diabetic nephropathy, focal segmental glomerulosclerosis, IgA nephropathy, membranous nephropathy, mesangial proliferative glomerulonephritis	39	Wnt/β‐Catenin		
		Chronic kidney disease (U)—diabetic nephropathy, focal segmental glomerulosclerosis, IgA nephropathy, membranous nephropathy, mesangial proliferative nephropathy, minimal change disease, amyloidosis nephropathy	52			
		Lupus nephritis (U)	32			
		Lupus nephritis (U)	65			
	miR‐200b	Chronic kidney disease (U)—IgA nephropathy, diabetic nephropathy, membranous nephropathy, focal segmental glomerulosclerosis, hypertensive kidney disease	50	ZEB1/2	↑ in steroid‐induced osteonecrosis of femoral head, epithelial ovarian cancer	^86^, ^175^, ^176^
	CD2AP mRNA	Chronic kidney disease (U)—diabetic nephropathy, membranous nephropathy, membranoproliferative glomerulonephritis	39	Nephrin	–	^177^
	hsa_circ_0036649	Chronic kidney disease (U)—IgA nephropathy, membranous nephropathy, minimal change disease, focal segmental glomerulosclerosis, diabetic nephropathy, hypertensive nephropathy, intracapillary proliferative glomerulonephritis, crescentic glomerulonephritis, minor glomerular abnormalities	164	NR	–	^178^

Abbreviations: B, blood; CKD, chronic kidney disease; CREBBP, CREB‐binding protein; HDAC5, histone deacetylase 5; IgA, immunoglobulin A; JAK/STAT, janus kinase/signal transducer and activator of transcription; MMP, matrix metalloproteinase; NF‐κB, nuclear factor kappa‐light‐chain‐enhancer of activated B cells; NR, not recorded; P, plasma; PI3K/AKT, phosphatidylinositol 3‐kinase/ protein kinase B; PTEN, phosphatase and TENsin homolog deleted on chromosome 10; Refs., reference; SMAD, small mothers against decapentaplegic; SNHG16, small nucleolar RNA host gene 16; SRSF6, serine/arginine‐rich splicing factor 6; TGF‐β1, transforming growth factor beta‐1; U, urine; Wnt, wingless‐related integration site; ZEB1/2, zinc finger E‐box‐binding homeobox 1/2.

The specificity of proposed EV‐based kidney fibrosis biomarkers need to be improved significantly prior to clinical implementation. Several EV‐based biomarkers are affected by other pathologies that are unrelated to kidney fibrosis. uEV‐based *miR‐21* positively correlates with interstitial fibrosis/tubular atrophy levels in glomerular disease, diabetic nephropathy and kidney transplants.^74^, ^75^ However, *miR‐21* is upregulated in uEVs of people with prostate,^76^ bladder^77^ and gastric^78^ cancer. *miR‐21* is also upregulated in serum, peritoneal and/or cervicovaginal fluid from people with solid organ malignancies, pulmonary and cardiac fibrosis; raising concerns regarding the specificity of *miR‐21* for kidney fibrosis.^79^ *miR‐29*s modulate tumour suppressor and oncogenic genes involved in cancer pathobiology.^80^ Different miR‐29 species (a, b, c) can vary in the serum of people with colorectal, bladder, hepatocellular and breast cancer; making it challenging to pinpoint positive or negative correlations between serum *miR‐29*s with cancer status.^80^ Urine *miR‐29a* was upregulated in people with bladder cancer but uEV *miR‐29a/c* were reduced in people with kidney fibrosis.^81^ Organ specificity may be addressed by assessing EVs from urine which are enriched for EVs from the kidneys.^73^, ^82^ There is currently no method to positively select for kidney EVs and the majority of commercial immuno‐affinity EV isolation kits select for tetraspanins (e.g., CD63, CD9, CD81). Current studies assessing EV‐based biomarkers include less than 250 participants which may not recapitulate the patient heterogeneity of real‐world multi‐comorbid clinical populations. This is particularly important as people with kidney disease have the highest mean number of comorbidities compared to patients treated by other specialties.^83^ Large‐scale clinical trials of multi‐comorbid cohorts are critical to evaluate the robustness of proposed EV‐based biomarkers for kidney fibrosis.

It becomes more complicated if EV‐based cargo can vary depending on the presence of superimposed acute kidney injury, the inciting event of the fibrogenic niche. uEV *miR‐29a* and *miR‐29c* is downregulated in people with kidney fibrosis due to CKD across five studies.^16^, ^84^, ^85^, ^86^ However, *miR‐29c* is upregulated in uEVs of deceased kidney transplant donors who may have a degree of acute kidney injury.^87^ Urine *miR‐29* is increased in people with acute kidney injury and early stages of diabetic kidney disease.^88^, ^89^ In tubular epithelial cells, *miR‐29* targets tropomyosin 1 to inhibit Wnt/β‐catenin‐mediated EMT.^90^ Enhanced *miR‐29* release during acute kidney injury may be a compensatory mechanism to prevent kidney fibrosis. Notably, *miR‐29* also plays protective roles in lung and liver fibrosis,^91^, ^92^ but pathogenic roles in heart fibrosis^93^—complicating its interpretation as a kidney fibrosis biomarker. These issues can be addressed by using multi‐biomarker panels which can include kidney‐ and disease‐specific biomarkers to improve specificity. In a study of people with diabetic kidney disease, a transcriptional score of 6 mRNAs (glutathione peroxidase 3 [GPX3], NADPH oxidase 4 [NOX4], methionine‐R‐sulphoxide reductase B [MSRB], methionine sulphoxide reductase A [MSRA], heat‐responsive protein 12 [HRSP12], crystallin alpha B [CRYAB]) better discriminated CKD progression compared to single markers.^94^ A compound biomarker for serum and blood EVs (serum: CD62P‐CD41b‐CD42a‐CD31; urine: CD105‐CD1c‐SSEA4‐CD133/1) correlated better with kidney recovery than individual markers in kidney transplant recipients.^95^ Furthermore, multi‐biomarker panels have in‐built redundancies to mitigate the impact of single biomarker outliers and provide information about multiple biological pathways in one test.

EV‐based biomarker selection into a kidney fibrosis panel should be informed by biological functionality as molecules that participate in the fibrogenic niche are more likely to be robust biomarkers. For example, EV‐based *miR‐150* and *miR‐19* have functional evidence supporting their roles in ECM deposition and tissue inflammation respectively.^32^, ^57^, ^58^ The majority of putative biomarkers for kidney fibrosis have been linked to kidney fibrosis events (Table 2). To date, the roles of circular RNAs, *hsa_circ_0008925* and *hsa_circ_0036649* in kidney fibrosis pathobiology have not been elucidated. Other non‐coding RNAs, such as lncRNA and transfer RNA (tRNA), have been implicated in kidney injury pathogenesis, with the increased accessibility of strand‐specific and long read sequencing.^96^, ^97^ lncRNA are highly organ‐ and disease‐specific making them favourable biomarker candidates.^98^ Further studies are required to investigate the potential of EV lncRNA and tRNAs for assessing kidney fibrosis.

The dynamicity of the fibrogenic niche highlights the need for reliable biomarkers to pinpoint underlying pathobiological processes in real‐time to facilitate mechanism‐driven therapy. Kidney fibrosis is heterogenous based on disease pathobiology and activity; processes at different stages of development; and balance of pro‐fibrotic to fibrolytic factors. The increased availability of targeted therapies (e.g., monoclonal antibodies, interfering RNAs) enable specific processes in the fibrogenic niche to be addressed—however, biomarker development has not kept up with these developments to guide therapy decisions. Current approaches to prevent CKD progression include the sequential trial and addition of therapies, often in order of discovery, based on published likelihood of success in clinical trials of similar cohorts. Robust biomarkers for active disease processes can streamline the selection of customised regimens to maximise therapeutic efficacy and minimise exposure to futile treatments. Considering that ‘time is kidney’, biomarker‐driven therapy selection is the edge required to accelerate commencement of efficacious anti‐fibrotic therapy and win the race against CKD. Further understanding of the role of EV cargos in kidney fibrosis pathobiology is essential to develop biomarkers that can not only indicate degree of kidney fibrosis but also, underlying mechanisms.

Development and validation of EV‐based biomarker panels for kidney fibrosis has lagged behind other urogenital diseases such as prostate cancer, where there is currently a Food and Drug Administration (FDA)‐approved uEV test (i.e., ExoDx Prostate Intelliscore) to triage need for prostate biopsy.^99^ Recently, Mercy BioAnalytics developed the Mercy Halo assay, a serum EV‐based test for ovarian cancer diagnosis which was granted Breakthrough Device Designation from the FDA for use in asymptomatic post‐menopausal women.^100^ The increased difficulty of distilling a unifying biomarker panel for kidney fibrosis can be attributed to the heterogeneity of fibrotic processes and diverse cell types involved. Current biomarker studies have primarily compared EVs from people with kidney fibrosis to healthy participants which raises confounding factors of primary kidney disease, proteinuria and haematuria—all of which impacts the specificity of putative biomarkers. Large‐scale biomarker studies are required with appropriate disease control groups across people with different kidney diseases, proteinuria/haematuria levels and comorbidities to identify EV‐based biomarkers that are sensitive and specific for kidney fibrosis. Putative biomarkers should also play roles in kidney fibrosis pathobiology to enhance specificity. The majority of EV biomarker studies for kidney fibrosis have focused on uEVs—however, future studies also need to assess blood EVs, particularly for people who are oligo‐anuric. EV biomarkers that are reduced in the urine may be increased in the blood in response to kidney fibrosis (e.g., *miR‐19*) highlighting the importance of concurrent urine and blood analyses to understand biomarker performance across multiple biofluids.

Roadblocks for clinical implementation of EV‐based biomarkers can be categorised into fundamental knowledge‐base, sample collection, normalisation, detection, data processing, assay development, approval and clinical aspects. Examples of roadblocks include: scant knowledge of EV origin and cargo composition, lack of standardised framework for collection and storage of bio‐samples, absence of validated markers for normalisation, limited capacity for single‐EV analysis and, scalability of EV isolation and detection technologies (Table 4). These roadblocks and proposed solutions are discussed in detail by the International Society of Extracellular Vesicles’ (ISEV) Urine Task Force.^101^ Initiatives by ISEV to address these roadblocks include Minimal Information for Studies of EVs (MISEV2023) guidelines^102^ to standardise reporting of EV studies, the release of a Urine Task Force position statement on uEVs and EV‐TRACK,^103^ a crowdsourcing knowledge‐base to centralise methodologies and results. Special interest groups within ISEV, such as the Genitourinary System EV (GUSEV) special interest group, address clinical translation roadblocks by increasing awareness of EVs, enhancing EV research quality and facilitating cross‐disciplinary collaborations. Putative EV biomarkers should also outperform existing kidney injury biomarkers such as KIM‐1, neutrophil gelatinase‐associated lipocalin (NGAL), ADMA and liver‐type fatty acid‐binding protein (L‐FABP) to overcome clinical inertia and enhance implementation.

## 5 EV‐BASED THERAPEUTICS FOR KIDNEY FIBROSIS

**FIGURE 4:**
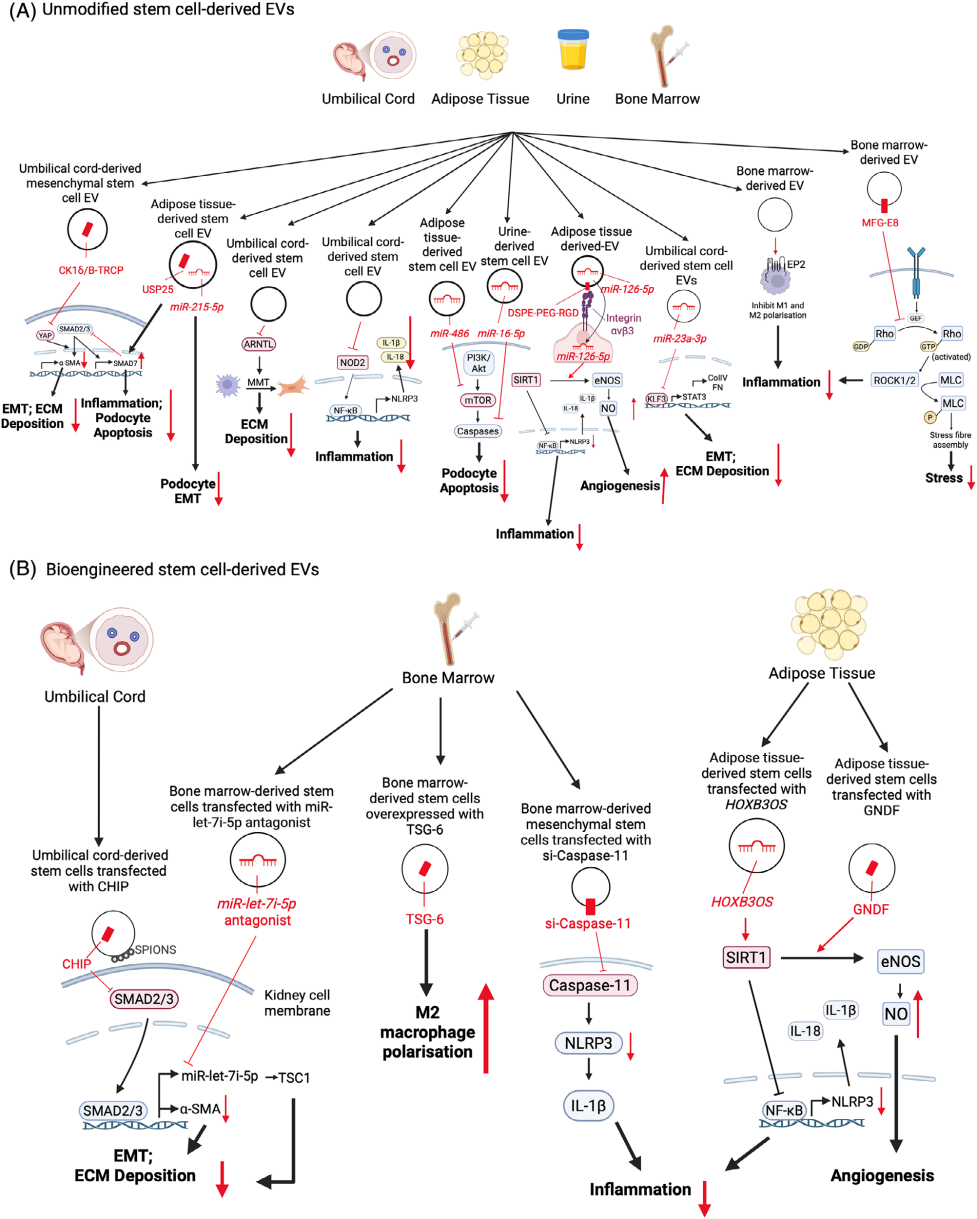
Mechanisms of proposed therapeutics for kidney fibrosis. CHIP, carboxyl terminus of Hsc70‐interacting protein; CK1δ/ B‐TRCP, CK1δ‐E3 and ubiquitin ligase β TRCP; col, collagen; DSPE‐PEG‐RGD, phosphatidylethanolamine 1,2‐distearoyl‐sn‐glycero‐3‐phosphoethanolamine‐N‐[RGD (polyethylene glycol)‐2000]; ECM, extracellular matrix; EMT, epithelial–mesenchymal transition; eNOS, endothelial nitric oxide synthase; FN, fibronectin; GNDF, glial‐derived neurotrophic factor; IL, interleukin; KLF3, Krüppel‐like factor 3; miR, microRNA; mTOR, mammalian target of rapamycin; NF‐κB, nuclear factor‐kappa B; NLRP3, nucleotide‐binding oligomerisation domain‐like receptor family pyrin domain‐containing 3; NO, nitric oxide; NOD2, nucleotide‐binding oligomerisation domain 2; PI3K/Akt, phosphatidylinositol 3‐kinase/protein kinase B; SIRT1, silent information regulator sirtuin 1; SMAD, suppressor of mothers against decapentaplegic; SPION, superparamagnetic iron oxide nanoparticles; STAT3, signal transducer and activator of transcription 3; TSC1, tuberous sclerosis 1; YAP, Yes‐associated protein; α‐SMA, alpha smooth muscle actin. Created in BioRender. Chiang, C. (2026) https://BioRender.com/9vvc10e.

EV‐based therapeutics for kidney fibrosis can be in the form of unmodified stem cell‐derived EVs and bioengineered stem cell‐derived EVs (Table 3 and Figure 4). Umbilical cord‐, adipose‐ and bone marrow‐derived mesenchymal stem cells are the most common source of EVs in therapeutics studies of kidney fibrosis. Umbilical cord‐derived mesenchymal stem cell EVs contain casein kinase 1δ/β‐transducin repeat‐containing E3 ubiquitin protein ligase (CK1δ/β‐TRCP), a kinase ubiquitin system which degrades transcription factor, Yes‐associated protein (YAP), to reduce SMAD2/3‐mediated α‐SMA expression.^104^, ^105^ Intravenous injection of these EVs (10 mg/kg/injection on days 6, 9, 12) into rats injured with UUO is shown to reduce α‐SMA expression and kidney fibrosis.^104^ Umbilical cord‐derived mesenchymal stem cell EVs also hold anti‐inflammatory potential in alleviating the progression of kidney fibrosis by inhibiting macrophage‐to‐myofibroblast transformation (MMT) in UUO mice.^106^ This is achieved by targeting aryl hydrocarbon receptor nuclear translocator‐like (ARNTL), a transcription factor that positively regulates MMT in kidney diseases.^107^ EVs secreted by umbilical cord‐derived stem cells also alleviate kidney fibrosis by suppressing inflammation via the nucleotide‐binding oligomerisation‐domain‐containing protein 2 (NOD2) pathway.

**TABLE 3:** Kidney fibrosis treatment with EVs.

Methods	Source of stem cells	Cargo	Targeted pathway	Effect	Refs.
EVs from unmodified cells^a^	Bone marrow	NR	EP2	Reduced polariton of M1 and M2 macrophages	^179^
		MFG‐E8	RhoA/ROCK	Reduce inflammation, oxidative stress, apoptosis, fibrosis	^180^
	Umbilical cord	NR	ARNTL	Inhibit macrophage–myofibroblast transition	^106^
		NR	NOD2	Suppress inflammation	^181^
		CK1δ/B‐TRCP	TGF‐β1/SMAD	Inhibit α‐SMA expression	^104^
		miR‐23a‐3p	KLF3/STAT3	Downregulate pro‐fibrotic genes (ColIV and FN)	^182^
	Adipose tissue	miR‐486	PI3K/AKT	Inhibit podocyte apoptosis	^14^
		miR‐215‐5p	ZEB2	Reduce podocyte EMT	^183^
		USP25	SMAD7	Reduce podocyte apoptosis and inflammation	^108^
		miR‐126–5p	SIRT1	Reduce vascular rarefaction	^122^ ^*^
	Urine	miR‐16‐5p	PI3K/AkT/caspase	Inhibit podocyte apoptosis	^184^
EVs from bioengineered cells^b^	Umbilical cord	CHIP	TGF‐β1/SMAD	Inhibit the expression of α‐SMA	^114^, ^e^
	Adipose tissue	HOXB3OS	SIRT1/NLRP3	Suppress inflammation	^121^, ^185^
		GNDF	SIRT1/eNOS	Promote angiogenesis at peritubular capillary and reduce oxidative stress by increasing the activity of eNOS	^115^
	Bone Marrow	si‐caspase‐11	Caspase 11	Suppress inflammation	^118^, ^186^
		miR‐let‐7i‐5p antagonist	TGF‐β1/SMAD	Inhibit miR‐let‐7b‐5p to increase TSC1 expression → inhibit EMT and ECM deposition	^116^
		TSG‐6	Multiple immune pathways	Promote M2 macrophage polarisation and regulatory T‐cell induction	^120^
Post‐modified EV^c^	Adipose tissue	DSPE‐PEG‐RGD	Integrin αvβ3	Dock via integrin αvβ3 on endothelial cells to deliver cargo	^122^, ^d^
	Umbilical cord	SPION	Magnetic targeting	Targets EVs to kidneys using magnetic field	^114^, ^e^

Abbreviations: ARNTL, aryl hydrocarbon receptor nuclear translocator‐like protein 1; CHIP, carboxyl terminus of HSC70 interacting protein; CK1δ/ B‐TRCP, casein kinase 1 delta/ beta‐transducin; ColIV, collagen IV; DSPE‐PEG‐RGD, phosphatidylethanolamine 1,2‐distearoyl‐sn‑glycero‐3‐phosphoethanolamine‐N‐[RGD (polyethylene glycol)‐2000]; ECM, extracellular matrix; EMT, epithelial–mesenchymal transition; eNOS, endothelial nitric oxide synthase; EP2, prostaglandin E2 receptor, EVs, extracellular vesicles; FN, fibronectin; MFG‐E8, milk fat globule‐epidermal growth factor‐factor 8, GNDF, glial cell line‐derived neurotrophic factor; miR, microRNA; mTOR, mechanistic target of rapamycin; NOD2, nucleotide‐binding oligomerisation domain protein 2; NR, not recorded; PI3K/ AKT, phosphatidylinositol 3‐kinase/ protein kinase B; Refs., references; SIRT1, sirtuin 1; SMAD, small mothers against decapentaplegic; SPION, superparamagnetic iron oxide nanoparticle, TGF‐β1, transforming growth factor beta‐1; TSC1, tuberous sclerosis complex‐1; TSG‐6, tumour necrosis factor‐α‐induced protein 6; USP25, ubiquitin‐specific protease 25; ZEB2, zinc finger E‐box‐binding homeobox 2; α‐SMA, alpha smooth muscle actin.

^a^ EVs from unmodified cells: EVs naturally produced by unmodified cells.

^b^ EVs from bioengineered cells: EVs naturally produced from cells modified to express cargo of interest.

^c^ Post‐modified EVs: EVs from unmodified or bioengineered cells that are modified after release.

^d^ EVs from unmodified cells that were subsequently post‐modified to carry DSPE‐PEG‐RGD.

^e^ EVs from bioengineered cells that were subsequently post‐modified to carry SPION.

In diabetic nephropathy, adipose‐derived mesenchymal stem cell EVs transfer *miR‐486*, *miR‐251*, *miR‐215‐5p* and ubiquitin‐specific protease 25 (USP25) which reduce podocyte injury and EMT.^14^, ^108^ *miR‐486* increases podocyte autophagy and reduces podocyte apoptosis which downregulates SMAD1 expression and inhibits mTOR activation.^14^ USP25 attenuates SMAD7 ubiquination in podocytes under higher glucose conditions, reducing apoptosis and cytokine (interleukin [IL]‐6, TNF‐α, IL‐1β and IL‐18) release.^108^ Human urine‐derived stem cells containing *miR‐16‐5p* improve podocyte viability and reduce podocyte apoptosis by downregulating the expression of caspase‐3 in diabetic nephropathy, while kidney‐derived mesenchymal stem cell EVs ameliorate peritubular capillary rarefaction in UUO mice via inhibition of inflammatory cell infiltration and endothelial–mesenchymal transition—ultimately reducing tubulointerstitial fibrosis.^109^ Bone marrow‐derived mesenchymal stem cell EVs have also been demonstrated to reduce kidney fibrosis in 5/6 nephrectomy rat, surgical bilateral ovariectomy rat, aristolochic acid–induced nephropathy mice and diabetic nephropathy rat models—although the causative cargos have not been identified.^110^, ^111^, ^112^, ^113^

EVs from bioengineered cells have been utilised as novel drug delivery systems. Cells can be transfected with RNA constructs to produce EVs enriched with specific cargos. Human umbilical cord mesenchymal stem cells transfected with a lentivirus vector encoding the carboxyl terminus of Hsc70‐interacting protein (CHIP) produce EVs enriched with this molecule—with EV transfer of CHIP to kidney tubular epithelial cells degrading SMAD2/3 to attenuate ECM deposition.^114^ The transfection of glial cell line‐derived neurotrophic factor (GDNF) into adipose mesenchymal stem cells via a lentivirus vector generates GDNF‐enriched EVs that trigger endothelial cell angiogenesis following injury and ameliorate peritubular capillary loss and tubulointerstitial fibrosis by activating the sirtuin 1/endothelial nitric oxide synthase (SIRT1/eNOS) signalling pathway.^115^ EVs from bone marrow‐derived mesenchymal stem cells transfected with a *miR‐let‐7i‐5p* antagomir using Lipofectamine 2000 inhibit *miR‐let‐7i‐5p* in TGF‐β1‐induced kidney tubular epithelial cells to increase expression of its target gene Tuberous sclerosis complex 1 (*TSC1*) and reduce ECM deposition and EMT.^116^ EVs from bone marrow‐derived stem cells transfected with small interfering RNA (siRNA) targeting caspase‐11 inhibit caspase 11 expression in CKD mice to reduce kidney fibrosis and restore partial kidney functions.^117^ Caspase‐11 is a positive regulator of kidney fibrosis that triggers inflammation via the cleavage of pro‐IL‐1β into active IL‐1β, a pro‐inflammatory cytokine.^118^ *miR‐23a‐3p* from EVs secreted by umbilical cord‐derived stem cells transfected with *miR‐23a‐3p* reduce kidney fibrosis in diabetic kidney disease mice by inhibiting the KLF3/STAT3 pathway.^27^ STAT3 is the central transcription factor for pro‐fibrotic genes, collagen IV and fibronectin.^119^ TNF‐α‐induced protein 6 (TSG‐6)‐enriched EVs from TSG‐6 overexpressing or indole‐3‐carbinol‐treated bone marrow‐derived mesenchymal stem cells promote M2 macrophage polarisation and regulatory T‐cell induction to suppress kidney fibrosis and inflammation after ischaemia–reperfusion injury.^120^ Adipose‐derived mesenchymal stem cells transfected with lncRNA *HOXB3OS* alleviate kidney inflammation in high glucose‐treated podocytes. *HOXB3OS*‐enriched EVs ameliorate inflammatory podocyte injury by enhancing the expression of SIRT1 that, in turn, inhibits transcription of proinflammatory NLRP3.^121^ Adipose‐derived mesenchymal stem cell EVs can also be modified with phosphatidylethanolamine 1,2‐distearoyl‐sn‑glycero‐3‐phosphoethanolamine‐N‐[RGD (polyethylene glycol)‐2000] (DSPE‐PEG‐RGD) to dock via integrin αvβ3 complex on endothelial cells to transfer *miR‐126–5p*. *miR‐126‐5p* modulates alkB homolog 5 (ALKBH5)‐mediated N6‐methyladenosine (m6A) modification of SIRT1 and reduces vascular rarefaction.^122^ Methods to suppress EV uptake in macrophages such as alvespimycin also attenuate mesangial expansion, inflammatory gene activation and proteinuria in diabetic mice.^123^ The effectiveness of strategies which suppress tubular epithelial cell EV uptake to reduce kidney fibrosis have not been tested.

Moving beyond preclinical testing, upscaling EV production and adherence to good manufacturing practices are essential for clinical implementation of EV therapeutics. Roadblocks to widespread EV therapeutics implementation include EV heterogeneity, sensitivity to small changes to production parameters, absence of standardised characterisation protocols, technological limitations and knowledge gaps on EV mechanisms of action. EVs, even from the same cell type, exhibit significant heterogeneity in size, density, viscoelasticity, molecular composition and function.^124^ Features ascribed to a bulk EV population cannot be ascribed to individual EVs. Population subtyping with single EV analytics is essential to adequately describe EV therapeutics. Whilst high‐throughput label‐free single‐EV analytics are available for physical properties such as size, charge, viscoelasticity; single‐EV molecular characterisation is predominantly reliant on immuno‐affinity‐based techniques (e.g., electron microscopy, super‐resolution microscopy, flow cytometry)—requiring marker pre‐selection. Commonly used label‐free methods such as next‐generation RNA sequencing and mass spectrometry require at least 10^5^–10^8^ EVs and are not able to handle the ultra‐low inputs from single EVs.^125^ Surface‐enhanced Raman spectroscopy offers a label‐free solution to single‐EV analysis by converting surface chemistry and molecular cargo into a single spectroscopic pattern. However, this technology is time consuming (90 min per 10–100 µL serum EVs) and not widely available.^125^ Cost‐effective, high‐throughput and high‐plex single EV analytics are urgently needed to improve characterisation of EV therapeutics to account for heterogeneity within and between batches.

Furthermore, EV cargos are highly sensitive to minor changes in production methods including passage number, culture method, culture medium and EV isolation.^126^, ^127^ Even in the same laboratory, EVs produced from the same mesenchymal stromal cell stocks exhibited divergent protein signatures on mass spectrometry with diverse immunomodulatory capacities.^128^ Conventional EV characteristics such as EV concentration and CD9, CD63, CD81, syntenin and calnexin concentrations are not able to distinguish active and inactive EV preparations. Screens of 372 miRNAs fail to identify any specific miRNAs to identify active or inactive EV preparations. These results highlight that intrinsic and extrinsic variabilities in manufacturing processes can effect subtle changes in EV characteristics which impact therapeutic efficacy. Variabilities in manufacturing processes can be largely mitigated by strict adherence to good manufacturing practices. Cell source heterogeneity can be addressed by utilising immortalised cells (e.g., HEK293) and maintaining consistent passage numbers across production batches. Further studies are needed to establish how variabilities in manufacturing practices impact EV characteristics and function to establish EV‐specific manufacturing and quality control guidelines.

The absence of standardised characterisation protocols for EV therapeutics makes it challenging to compare between EV products. This issue is underpinned by modest understanding of EV mechanisms of action and limitations in EV characterisation tools. EVs are highly heterogenous and EVs from a single cell source can contain over 10,000 RNAs, over 100 proteins and over 400 lipid species.^124^, ^129^ The biological function of only a fraction of these constituents are known making it difficult to protocolise ‘essential’ defining features of EV therapeutics. Current EV characterisation commonly includes size, concentration, direct visualisation, EV marker (e.g., CD9, CD63, CD81) and contaminant quantification. Nanoparticle tracking analysis, tuneable resistive pulse sensing and flow cytometry offers high throughout testing for these parameters. Direct visualisation, whilst able to provide discrete measurements for size and markers, is low throughput, labour‐intensive and requires specialised equipment. Omics technologies can confirm the consistency of diverse EV constituents between different batches. Borrowing from the cell therapies field, functional assays can test the potency and efficacy of EV therapeutics in vitro mitigating the need to understand the function of every EV constituent. Standardisation of a matrix of functional assays which verifies product efficacy/potency in vitro is essential for the quality control of EV therapeutics.^128^, ^130^, ^131^

Other challenges for EV‐based therapeutics include kidney targeting and maximising EV half‐life to ensure cargo delivery to site of action. Intravenously injected EVs accumulate in the liver and modifications to the EV surface are required to enhance EV delivery to the kidneys.^132^ Post‐modifications such as DSPE‐PEG‐RGD binding Integrin αvβ3,^122^ LTHVVWL binding KIM‐1^133^ and superparamagnetic iron oxide nanoparticle (SPION) enabling magnetic targeting^114^ have increased EV delivery to the kidneys. Kidney targeting peptides such as (KKEEE)^3^K and G3‐C12 which binds low‐density lipoprotein receptor‐related protein 2 (LRP2)^134^ and CLPVASC peptide have primarily been used with EV mimetics.^135^ Targeting kidney antigens with antibodies or fragment antigen binding (Fab) fragments may also facilitate EV delivery to the kidneys.^136^ The majority of kidney targeting strategies focus on tubular cells and strategies are needed to target other cells which participate in kidney fibrosis. EVs are rapidly cleared from circulation via the mononuclear phagocyte system and intravenously administered EVs have a half‐life of 2–45 min.^137^ Strategies to enhance EV half‐life involve surface modifications to prevent EV uptake by macrophages and monocytes. These strategies include overexpression of CD47 on EV surface; association of albumin binding domains (ABDs) to EV‐associated proteins (CD63/CD9/CD81/Lysosome‐associated membrane protein 2 [LAMP2]) or lipids (dioleoylphosphatidylethanolamine [DOPE], cholesterol, ceramide) and incorporation of synthetic polymers (PEGylation, POxylation).^138^, ^139^, ^140^, ^141^ ABD‐CD63 modification of EVs led to 10 fold increase in circulating EV count at 270 min compared to unmodified EVs. However, circulating ABD‐CD63 EVs at 270 min constituted less than 0.1% of injected ABD‐CD63 EVs.^138^, ^139^ Anti‐PEG IgG and IgM antibodies are present in 6%–24% healthy individuals, highlighting the potential for hypersensitivity reactions to PEGylated EVs.^142^ Non‐immunogenic strategies that substantially increase EV targeting of different kidney cells and enhance EV persistence in circulation are urgently needed to improve EV therapeutic delivery.

Clinical translation of EV‐based therapeutics lag behind those for cellular therapies, despite extensive preclinical evidence for efficacy and safety (Table 4). EVs from a range of cellular sources including mesenchymal stem cells,^143^, ^144^, ^145^, ^146^, ^147^ dendritic cells,^148^, ^149^ HEK293 cells,^150^, ^151^, ^152^ platelets^153^, ^154^ and cardiac progenitor cells^155^ have been studied in cancer, CKD, Crohn's Disease, COVID‐19, alopecia, cardiac disease and healthy cohorts with minimal treatment‐related adverse events. Tested routes of EVs administration included subcutaneous, intradermal, intravenous, intra‐arterial, nebulised, intracoronary, topical, oral and sublingual; with intravenous being the commonly tested. Intravenous EV doses ranged from 3.0 × 10^9^ EVs/kg/dose to 5.2 × 10^10^ EVs/kg/dose with up to 6 doses administered.^147^, ^152^ Further studies are required to establish optimal EV dosage and route of administration for CKD treatment. Completed studies have reported promising efficacy data however, these studies evaluated small sample sizes (*n* = 5–91) and the majority of studies (6/9, 67%) had no placebo or standard‐of‐care comparator arm. These results highlight the critical need for large Phase 3 randomised controlled trials to assess the efficacy of EV therapies. There has only been one study of EVs in CKD where two doses of 100 µg/kg mesenchymal stem cell EVs (or placebo) administered to 40 people with CKD led to improved serum creatinine and eGFR at 12 weeks but not 1 year.^143^ There was increased levels of immunomodulatory cytokines (TGF‐β1, IL‐10) and reduced levels of TNF‐α at 12 weeks.^143^ Further adequately powered studies with longer treatment duration and follow‐up are urgently needed to realise the potential of EV therapeutics for kidney fibrosis.

**TABLE 4:** Clinical trials of EVs for which there is safety data available for investigational product (completed and ongoing).

Study	EV product	Administration	Dose	Disease	Outcome	Refs.
Phase I, *n* = 15, 2005	Autologous monocyte‐derived DCs EVs loaded with MHCI and MHCII	Subcutaneous and intradermal	1.3 × 10^13^–4.0 × 10^13^ EVs/dose weekly for 4 weeks; no SOC/placebo arm	Metastatic melanoma	Three stable disease, one partial response, 11 progressive disease; maximal tolerated dose not achieved	^148^
Phase II, *n* = 22, 2015	Interferon‐gamma matured DC EVs loaded with MHCI and MHCII restricted cancer antigens	Intradermal	1.26 × 10^13^–1.67 × 10^14^ EVs/dose weekly for 4 weeks (*n* = 22) then 2 weeks off then fortnightly for 12 weeks (*n* = 10) then 2 weeks break then 3 weekly until progression or EV unavailability (*n* = 7); no SOC/placebo arm	NSCLC	Did not achieve primary endpoint of >50% patients with progression‐free survival at 4 months, seven patients stabilisatio*n* > 4 months, no objective tumour response; no treatment‐related death, one patient grade 3 hepatotoxicity	^149^
Phase II/III RCT, *n* = 40, 2016	Cord‐blood MSCs EVs	Intravenous, intra‐arterial (renal artery)	100 µg/kg/dose IV then 1 week later 100 µg/kg/dose IA; placebo arm received IV saline but not intra‐arterial injections	CKD	Improved serum creatinine and eGFR at 12 weeks but not 1 year; increased levels of serum TGF‐β1, IL‐10 and reduced levels of serum TNF‐α at 12 weeks; no significant adverse events up to 1 year	^143^
Phase I, *n* = 5, 2022	Umbilical cord MSCs EVs	Injection 2–3 mm into tissue around fistula tract	Single injection of 5 mL EV solution (concentration not reported); no SOC/placebo arm	Refractory peri‐anal CD fistulas	At 6 months, three patients with complete closure, one no improvement, one active discharge	^144^
Phase II RCT, *N* = 102, 2023 NCT04493242	Bone marrow MSCs EVs (ExoFlo)	Intravenous	0.9 × 10^12^ EVs/dose or 1.2 × 10^12^ EVs/dose on day 1 and 4; placebo controlled	COVID‐19 ARDS	Reduced 60‐day mortality and increased ventilation‐free days in higher dose ExoFlo group compared to placebo; no treatment‐related adverse event	^145^
Phase II, *n* = 91, 2024 NCT04902183	HEK293 cell EVs loaded with CD24	Nebulised	10^9^–10^10^ EVs/dose daily for 5 days; retrospective propensity score matched control cohort	COVID‐19 infection	At day 7, 83.7% had Improvement in respiratory rate, 64% had improvement; no treatment‐related adverse events	^150^, ^151^
Phase I/II, *n* = 12, 2024 NCT05658094	Placental MSCs EVs	Frontal, parietal, temporal intradermal injection	10^11^ EVs/dose every fortnight up to a maximum of 4 doses; no SOC/placebo arm	Alopecia	Improvement in hair density (hairs/cm^2^) at 3 and 6 weeks	^146^
Phase I RCT, *n* = 18, 2025 NCT05843799	HEK293 cell EVs loaded with lκBα (ILB‐202)	Intravenous	Single dose of .3 × 10^10^ EVs/kg, 1.2 × 10^10^ EVs/kg or 5.2 × 10^10^ EVs/kg; placebo controlled	Healthy	Mild increase NK cell counts, grade 1 neutropenia, modulation of NF‐κB pathways	^152^
Phase I, *n* = 9, 2025 NCT04327635	Platelet‐derived EVs (PEP)	Intracoronary infusion distal to newly placed stent	Single dose of 10 mL of 5%, 10% or 20% PEP; no SOC/placebo arm	Coronary artery disease	Results not yet available, marked as completed on 22 October, 2025 on clinicaltrials.gov	^153^
Phase I, *n* = 8, unknown status, NCT04664738	Platelet‐derived EVs (PEP)	Direct to skin donor wound	Single dose of 10% PEP, 20% PEP or 20% PEP/fibrin sealant from pooled human plasma; no SOC/placebo arm	Skin graft donor site wound	In a case report, 4× weekly topical applications of PEP mixed with collagen to a non‐healing scalp wound as associated with wound healing	^154^
Phase I, *n* = 60, unknown status, NCT04698447	EVs from *Citrus limon* juice (CitraVes)	Sublingual	1000 mg/day for 3 months; placebo controlled	Healthy	Preliminary data from 20 participants showed reduction in waist circumference at 4 and 12 weeks vs. baseline, reduction in LDL‐C at 12 vs. 4 weeks	^187^
Phase I, *n* = 12, ongoing, NCT05774509, 2022‐001844‐75 (EU)	Cardiac progenitor cells differentiated from iPSCs‐derived EVs	Intravenous	2.0 × 10^10^ EVs/kg every 3 weeks for 9 weeks or 4.0 × 10^10^ EVs/kg every 3 weeks for 9 weeks; no SOC/placebo arm	Non‐ischaemic DCM	For first participant, no severe adverse events, no post‐treatment ventricular arrhythmias, no inflammation (reduced CRP), no donor‐specific antibodies at 28 days; improved LVEDV, LVESV, LVEF	^155^
Phase III RCT, *n* = 970, ongoing, NCT05354141	Bone marrow MSCs EVs (ExoFlo)	Intravenous	1.2 × 10^12^ EVs/dose daily for 5 days; placebo controlled	COVID‐19 ARDS	3 × 5d courses associated with reduced lung inflammation and fibrosis in a case report, no adverse events	^147^

Abbreviations: ARDS, acute respiratory distress syndrome; CD, Crohn's disease; CKD, chronic kidney disease; COVID‐19, coronavirus disease 2019; CRP, C‐reactive protein; DC, dendritic cells; DCM, dilated cardiomyopathy; eGFR, estimated glomerular filtration rate; EV, extracellular vesicles; HEK293, human embryonic kidney 293; IL, interleukin; iPSC, induced pluripotent stem cells; LDL‐C, low‐density lipoprotein C; lκBα, Inhibitor of kappa‐light‐chain‐enhancer of activated B cells alpha; MHC, major histocompatibility complex; MSC, mesenchymal stem cells; *n*, sample size; NF‐κB, nuclear factor‐kappa B; NK, natural killer cell; NSCLC, non‐small cell lung cancer; SOC, standard‐of‐care; TGF‐β1, transforming growth factor beta 1; TNF‐α, tumour necrosis factor alpha.

## 6 CONCLUSIONS

EVs are essential cell‐to‐cell messengers in the coordination of kidney fibrosis processes within the fibrogenic niche. EV cargos orchestrate transitions from tissue injury and inflammation to vascular rarefaction, atrophy and ECM deposition. However, current studies have only revealed the tip of the iceberg. Further studies are required to phenotype the range of molecules in heterogenous EVs and map their mechanistic pathways to identify sensitive and specific biomarkers for kidney fibrosis; and fibrosis‐specific targets to tackle CKD. Technological advances are urgently needed to increase throughput and sensitivity of label‐free molecular profiling for single‐EV multi‐omics analysis. Spatial multi‐omics technologies can further understanding of EV function in situ. Improvements in EV engineering are required to enhance EV delivery to the kidney and EV half‐life. Concerted international efforts are urgently needed to standardise EV isolation, characterisation, production and protocol reporting. ISEV has led this charge with the development of MISEV2023 guidelines, consensus state‐of‐the‐art statements and EV‐TRACK. Importantly, standardised protocols need to be underpinned by an in‐depth understanding of how EV characteristics and upstream methodological aspects affect biomarker readouts and EV therapeutic product.

## AUTHOR CONTRIBUTIONS

Monica Suet Ying Ng and Chin‐Ya Sophie Chiang conceptualised study. Monica Suet Ying Ng and Chin‐Ya Sophie Chiang wrote original draft. Monica Suet Ying Ng, Helen Grania Healy and Andrew J. Kassianos reviewed manuscript. Monica Suet Ying Ng and Chin‐Ya Sophie Chiang prepared figures in manuscript. All the authors have read and agreed to the published version of the manuscript.

## CONFLICT OF INTEREST STATEMENT

Monica Suet Ying Ng has received consulting fees from Boehringer Ingelheim for work unrelated to this publication.

## ETHICS STATEMENT

The authors have nothing to report.

## CONSENT FOR PUBLICATION

The authors have nothing to report.

## Data Availability

Data sharing is not applicable to this article as no datasets were generated or analysed during the current study.
